# Towards Robot Scientists for autonomous scientific discovery

**DOI:** 10.1186/1759-4499-2-1

**Published:** 2010-01-04

**Authors:** Andrew Sparkes, Wayne Aubrey, Emma Byrne, Amanda Clare, Muhammed N Khan, Maria Liakata, Magdalena Markham, Jem Rowland, Larisa N Soldatova, Kenneth E Whelan, Michael Young, Ross D King

**Affiliations:** 1 Computational Biology Group, Department of Computer Science, Penglais Campus, Aberystwyth University, Aberystwyth, SY23 3DB, UK; 2 Institute of Biological, Environmental and Rural Sciences, Aberystwyth University, SY23 3DD, UK; 3 School of Engineering and Information, Middlesex University, NW4 4BT, UK

## Abstract

We review the main components of autonomous scientific discovery, and how they lead to the concept of a Robot Scientist. This is a system which uses techniques from artificial intelligence to automate all aspects of the scientific discovery process: it generates hypotheses from a computer model of the domain, designs experiments to test these hypotheses, runs the physical experiments using robotic systems, analyses and interprets the resulting data, and repeats the cycle. We describe our two prototype Robot Scientists: Adam and Eve. Adam has recently proven the potential of such systems by identifying twelve genes responsible for catalysing specific reactions in the metabolic pathways of the yeast *Saccharomyces cerevisiae*. This work has been formally recorded in great detail using logic. We argue that the reporting of science needs to become fully formalised and that Robot Scientists can help achieve this. This will make scientific information more reproducible and reusable, and promote the integration of computers in scientific reasoning. We believe the greater automation of both the physical and intellectual aspects of scientific investigations to be essential to the future of science. Greater automation improves the accuracy and reliability of experiments, increases the pace of discovery and, in common with conventional laboratory automation, removes tedious and repetitive tasks from the human scientist.

## Review

### Towards the full automation of scientific discovery

A Robot Scientist encompasses a combination of different technologies: computer controlled scientific instruments, integrated robotic automation to link the instruments together, a computational model of the object of study, artificial intelligence and machine learning to iteratively create hypotheses about a problem and later interpret experimental results (closed-loop learning), and the formalisation of the scientific discovery process. We show how these elements come together to create an automated closed-loop learning system: a Robot Scientist.

Automation in all its forms has played an integral role in the development of human society since the 19th century. The advent of computers and computer science in the mid-20th century made practical the idea of automating aspects of scientific discovery, and now computing is playing an increasingly prominent role in the scientific discovery process [1,2]. Experimental scientists use computers for instrument control, data acquisition and data analysis, and the functionality available on scientific instrumentation controlled by computers is improving rapidly. In addition, an increasing number of scientists no longer conduct physical experiments, instead using simulation or data-mining to discover new knowledge from existing data [3]. Artificial intelligence (AI) has been used in an attempt to automate some of the intelligent aspects of the scientific discovery process still predominantly carried out by human scientists. Some examples of systems using AI components follow:

DENDRAL was an AI program developed in the 1960s that used background knowledge of chemistry to analyse experimental mass spectra data. It used heuristic search to determine solutions for the chemical structures responsible for the spectra, and was the first application of AI to a problem of scientific reasoning. This version became known as Heuristic-DENDRAL. A variant called Meta-DENDRAL followed, and was the first expert system for scientific hypothesis formation. It took a set of possible chemical structures and corresponding mass spectra as input, and inferred a set of hypotheses to explain correlation between some of the proposed structures and the mass spectrum. This information was then used to describe the knowledge that Heuristic-DENDRAL could utilise in its search for suitable structures [4].

AM was an Automated Mathematician, a heuristic artificial intelligence program that modelled mathematical discovery in the mid 1970s [5]. It was said to have discovered numbers, prime numbers and several interesting mathematical conjectures. This system later evolved into EURISKO, developed in the late 1970s, which was more flexible in that it could be applied to other task domains. EURISKO was used successfully, for example, in optimising the design of integrated circuits for microchips [5].

KEKADA was a another heuristic based system that could develop hypotheses and plan experiments, searching for surprising phenomena [6]. Kulkarni and Simon used this system to model the discovery of the urea synthesis pathway by Krebs. However, KEKADA had limited background knowledge when compared to human scientists, and like AM and EURISKO, needed more heuristics in order to continue its discoveries.

BACON [7], ABACUS [8], Fahrenheit [9] and IDS [10] were automated data driven discovery systems that could discover scientific laws as algebraic equations. They relied on data being entered by the experimenter, or on simulation of experiments. More recently, another example of a data driven system uses iterative cycles of algorithmic correlation to extract natural laws of geometric and momentum conservation, using data captured from motion-tracking experiments [11].

Most intelligent scientific discovery programs still do not 'close the loop' (feeding their results back into their experimental models) and do not design or execute their own experiments. Now, with recent advances in hardware and technology, the limitations of being unable to run experiments are diminishing. For example, microfluidics ('lab-on-a-chip') based approaches to experimentation may soon allow small controllable biological experimental systems to be linked to automated scientific discovery [12,13]. We look forward to these advances in technology promoting future developments in automated scientific discovery. Integrated robotic systems are now capable of carrying out highly complex scientific discovery processes [14-18]. The majority of such automation is designed to perform experiments, previously conducted manually, in a more efficient, reliable and accurate manner than a human could ever achieve. Automation also enables previously impractical experiments to be carried out (such experiments may involve dangerous chemicals, pathogenic organisms, numerous assays or process steps, or need frequent measurements over long periods). With this type of system a human scientist typically sets-up the automated system to perform a sequence of processes, the system then executes the various steps automatically, and finally the human analyses the results. Any computation involved is usually connected with running the system, or with data management, visualisation and analysis. Complex databases are required to manage scientific data and knowledge and, as a result, science increasingly depends on efficient information management and manipulation [19].

Computational models or simulations have been used to give insight into how complex systems work; they represent the current state of understanding, provide a basis for predictions, and also have the benefit of being relatively cheap to execute. Models can be perturbed by making computational modifications to external conditions or to the architecture of the models themselves, then tested against acquired data. Such models can be constructed manually using human knowledge (e.g. [20]) or be automatically derived from experimental data (e.g. [11]).

The formal recording of scientific experimental data and meta-data can help towards creating better models, as well as facilitating the easier reuse of that data. Recording scientific data and meta-data in formal languages can provide complete, accurate, and detailed descriptions of why experiments were performed, how they were carried out, what the observed and derived results were, and how these results were interpreted. A well implemented formalisation provides the transparency required for science, allowing others to understand exactly why, as well as how an experiment was done, and provides all the essential information required to repeat that experiment.

To fully automate the scientific discovery process, computers also need to be able to create the initial hypotheses that define the reasons for carrying out the experiments, and then to be capable of learning from the results. Deduction, induction and abduction are types of logical reasoning used in scientific discovery [21]. *Deduction *enables the inference of valid facts from existing known facts and rules, *induction *enables the inference of hypothesised rules from known facts, and *abduction *enables the inference of hypothesised facts from known facts.

The full automation of science requires 'closed-loop learning', where the computer not only analyses the results, but learns from them and feeds the resulting knowledge back into the next cycle of the process [22]. Computational closed-loop learning systems have certain advantages over human scientists: their biases are explicit, they can produce full records of their reasoning processes, they can incorporate large volumes of explicit background knowledge, they can incorporate explicit complex models, they can analyse data much faster, and they do not need to rest.

### The Robot Scientist concept

**Figure 1:**
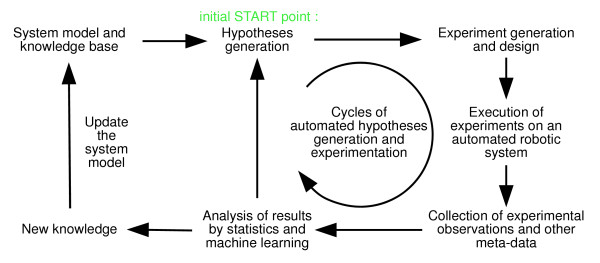
**Hypothesis-driven closed-loop learning**. Diagram showing how iterative cycles of hypothesis-driven experimentation allow for the autonomous generation of new scientific knowledge.

The combination of computational methods, automated instruments, closed-loop learning, advanced laboratory robotic systems and formal logical expression of information leads to the concept of a 'Robot Scientist' [23]. A Robot Scientist uses techniques from the field of artificial intelligence to carry out cycles of experimentation on a laboratory robotic system. It automatically generates hypotheses from the available background knowledge and model(s), designs physical experiments to test these hypotheses, carries out the experiments on a laboratory robotic system, and then analyses and interprets the results (see Figure 1). Because computers are involved throughout, it is possible to explicitly capture every detail of the scientific discovery process: goals, hypotheses, results, conclusions, etc. Moreover, in addition to all the direct experimental data there is also a wealth of useful meta-data that can be captured, such as environmental conditions, detailed experiment content layout information, and instrument settings, protocols and runtime logs. These meta-data can be especially important when studying complex biological systems where the specifics of the environment can have such a large effect on results.

### Robot Scientist prototypes

Here we describe our two prototype Robot Scientists, 'Adam' and 'Eve'. Adam has already proven itself by discovering new knowledge [24], whilst Eve is still under development. Both robots are designed to carry out biomedical scientific research.

#### A Robot Scientist to study yeast metabolism - 'Adam'

**Figure 2:**
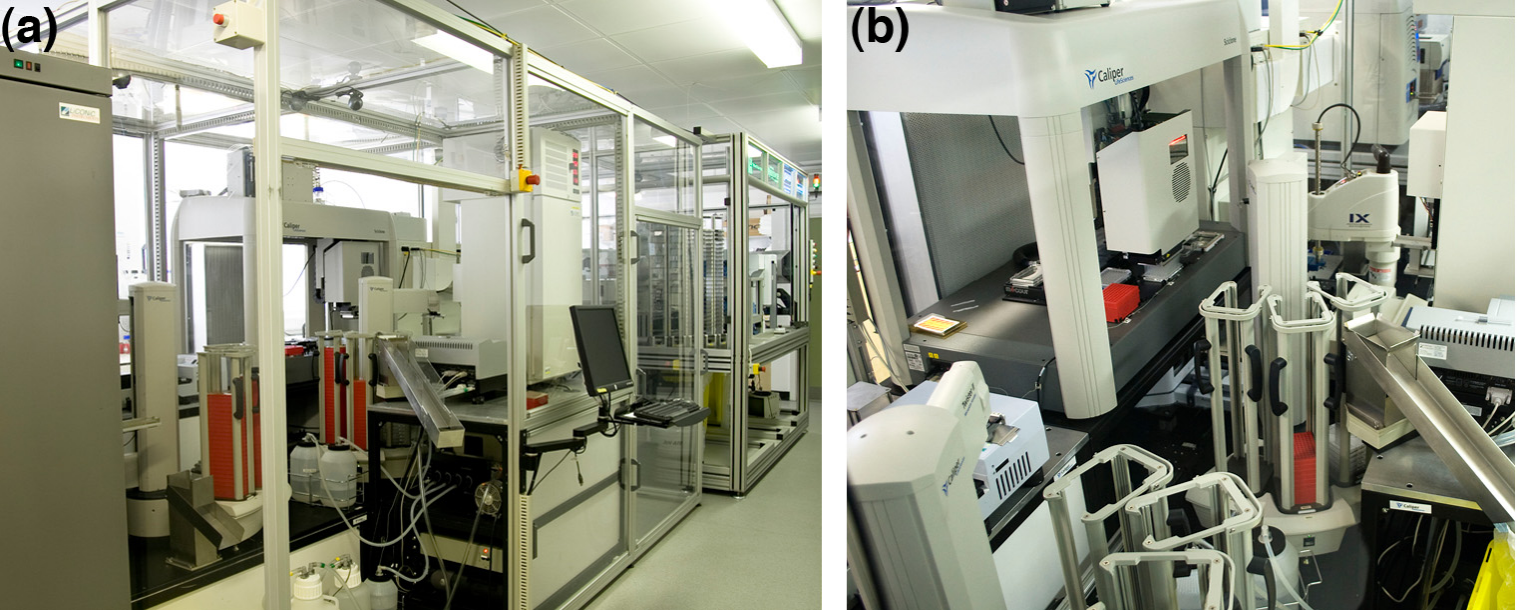
**Adam's laboratory robotic system**. (a) An external view of Adam's laboratory robotic system, also showing Eve's on the far right, and (b) a view looking down through the middle of Adam's robotic system, again with Eve's beyond.

Our first prototype Robot Scientist, Adam, was physically commissioned at the end of 2005 (see Figure 2). It was designed to carry out microbial growth experiments to study functional genomics in the yeast *Saccharomyces cerevisiae*, specifically to identify the genes encoding 'locally orphan enzymes'. A locally orphan enzyme is an enzyme that is known to exist in an organism, but where the corresponding gene is as yet unidentified (definition agreed with Yannick Poullot and Peter D. Karp, who defined the term 'orphan enzyme' which has a slightly different meaning, see [25]). Adam uses a comprehensive logical model of yeast metabolism (based on the Forster iFF708 model), coupled with a bioinformatic database (Kyoto Encyclopaedia of Genes and Genomes - KEGG) and standard bioinformatics homology search techniques (PSI-BLAST and FASTA) to hypothesise likely candidate genes that may encode the locally orphan enzymes. This hypothesis generation process is abductive. There were two types of hypothesis generated. The first level links an orphan enzyme, represented by its enzyme class (E.C.) number, to a gene (ORF) that potentially encodes it. This relation is expressed as a two place predicate where the first argument is the ORF and the second the E.C. number. An example hypothesis at this level is:

encodesORFtoEC('YBR166C', '1.1.1.25')

The second level of hypothesis involves the association between a deletant strain, referenced via the name of its missing ORF, and a chemical compound which should affect the growth of the strain, if added as a nutrient to its environment. This level of hypothesis is derived from the first by logical inference using our model of yeast metabolism. An example of such a hypothesis is: affects growth('C00108','YBR166C'), where the first argument is the compound (names according to KEGG) and the second argument is the deletant strain. More examples of Adam's hypotheses can be found on our website (see appendix note 1). Adam then designs the experimental assays required to test these hypotheses for execution on the laboratory robotic system. These experiments are based on a two-factor design that compares multiple replicates of deletant strains with and without metabolites compared against wild type strain controls with and without metabolites. Full details of the experiment design process (such as how suitable metabolites were chosen) can be found on the Robot Scientist website (see appendix note 1).

**Figure 3:**
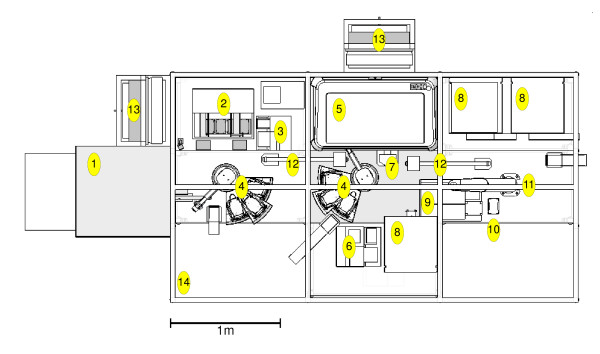
**Plan diagram of Adam's laboratory robotic system**. Layout diagram of Adam's laboratory robotic system, comprising: [1] Liconic STR602 freezer, [2] Caliper Presto liquid handler, [3] Thermo 384 multidrop, [4] two Caliper Twister II robot arms, [5] Caliper Sciclone i1000 liquid handler, [6] Bio-Tek ELx405 plate washer, [7] Agilent (Velocity 11) VSpin plate centrifuge, [8] three Liconic STX40 incubators, [9]> two Molecular Devices Spectramax 190 plate readers, [10] Variomag plate shaker, [11] IAI Corporation Scara robot arm, [12] two pneumatically actuated plate slides, [13] two high efficiency particulate air (HEPA) filters, and [14] aluminium and rigid transparent plastic enclosure. There are also four computers controlling the robotics, plus a networked computer server which runs all the other code vital to Adam's function: the metabolism model, bioinfomatics, hypothesis generation, experiment planning, results relational database, data analysis etc. (not shown).

Adam's robotic system comprises various automated laboratory instruments served by three robotic arms (see Figure 3). Experiments are created by combining the planned yeast strains, metabolites and defined growth medium solutions in SBS (Society for Biomolecular Screening) format microtitre plates, at medium to high throughput using a number of conventional liquid handlers (one of which is capable of aspirating or dispensing different volumes in 96 wells simultaneously - see appendix note 2). Adam is capable of creating up to 1000 individual experiments in a day, with a typical experiment running for 4 days.

The measurements observed by the system are optical density measurements taken at 595 nm recorded by the two microtitre plate readers (see appendix note 3), which when plotted over time become graphs that act as a proxy for cellular growth and indicate phenotype. The growth curves are smoothed, after which biologically significant parameters are extracted and statistically analysed to determine whether the original hypotheses have been confirmed or refuted. Scientific knowledge gained from this process is used to update the model of yeast metabolism. Full details about all these processes can be found on the Robot Scientist website (see appendix note 1).

The yeast *S. cerevisiae *is extensively studied as a model for eukaryotic cells. Yeast has a small physical size and fast generation time which makes it suitable for high-throughput experimentation, plus the growth curve results are relatively easy to observe and highly sensitive to changes in genotype and environment. There already exists a vast amount of information about yeast, including a detailed (but still incomplete) logical model of its metabolic pathways [26]. In Adam this model was used both as the basis for forming hypotheses, and also when designing the experiments required to test these hypotheses [20].

##### Adam's software

Adam is intended to be fully automated, with human intervention required only to supply library strain stocks and consumables. As such, Adam includes a collection of software components that together allow the system to perform cycles of experimentation.

In Adam's early work, the Inductive Logic Programming program C-Progol 5 [27,28] was used to automatically infer hypotheses for the investigation of aromatic amino acid metabolism. The inference was a restricted form of Abductive Logic Programming [29], where an incomplete background theory and experimental observations were used to infer facts concerning gene function. A logic program corresponding to the Aromatic Amino Acid Biosynthesis pathway of *S. cerevisiae *was used as the background theory and the inferred facts matched an ORF from yeast to a catalysing enzyme. The hypotheses completed the background theory by rediscovering the missing ORF/enzyme relations.

For Adam's most recent hypothesis generation work we have a detailed logical computer model of the metabolic reaction pathways in yeast (written in Prolog), from which locally orphaned enzymes are identified. The bioinformatics method of hypotheses generation attempts to use sequence similarity techniques to identify likely candidates for the ORFs that catalyse these reactions, thereby allowing the Robot Scientist to discover novel biology. The method is described by the following steps:

1. Identify Enzyme Commission (E.C.) numbers corresponding to enzymes which participate in yeast metabolism but have no known ORF assigned to them.

2. For each E.C. number find the ORFs in other organisms that code for that enzyme. Use all organisms from the KEGG genome database for this search. Collect all amino acid sequences for these ORFs. These are known as the 'query sequences'.

3. For each query sequence use sequence similarity search (PSI-BLAST or FASTA) to identify the most similar sequences/ORFs in *S. cerevisiae*.

4. A single hypothesis is the mapping of one *S. cerevisiae *ORF to one E.C. class - e.g. YER152C → 2.6.1.39. There are typically many hypotheses for each enzyme class.

Experiment design code then uses the system model to generate biological experiment plans involving deletant strains and metabolite solutions to test the hypotheses, creating microplate layouts using Latin-square design to improve the detection of quantitatively small differences above the background noise. The microplate layouts and related liquid handler volume files are passed to the robotic system control software, that executes the experiment plans. The resulting growth curve data is processed using algorithms based on cubic splines to fit, smooth and de-noise the curves, and then to extract biologically significant parameters such as growth rate and lag time. The parameters from multiple experiment replicates are analysed using machine learning (random forests [30]) to obtain statistically significant results that can be used to either confirm or refute hypotheses, potentially resulting in new scientific knowledge that can be used to update the system model. The cycle can then repeat with further hypothesis generation.

Finally, there is a comprehensive custom-made relational database that stores all the data and meta-data generated throughout the various stages (MySQL).

Further details describing all the software and informatics can be found online at our website (see appendix note 1).

##### Adam's results

Adam conceived 20 hypotheses concerning the identity of genes encoding 13 locally orphan enzymes in *S. cerevisiae*. Adam tested all these hypotheses on its robotic system and was able to confirm by experimentation, with a high degree of confidence, the correctness of 12 of them [24]. Conventional manual biological experiments were performed to verify 3 of these conclusions, and additional detailed literature searches revealed evidence supporting a further 6 more. Subsequent comparative genomics also indicated a number of possible reasons why the identities of some of the genes encoding these locally orphan enzymes had remained unknown for so long: there appear to have been gene duplication events with retained overlapping functions, a number of the enzymes appear to catalyse more than one associated reaction, and some of the functional annotations in the existing literature are incorrect. Adam's use of bioinformatic and quantitative phenotypic analyses were needed to deconvolve this functional complexity. Interestingly, Adam also came to an incorrect conclusion regarding one of its original 20 hypotheses which highlights a weakness in its system model. This was because the hypothesised gene candidate YIL033C was predicted to be a glutaminase enzyme, and Adam confirmed this activity experimentally by performing assays involving 11 metabolites predicted to have differential effect on a glutaminase deletant. However, it transpires that YIL033C has a cAMP-dependent protein kinase sub-unit which is involved in regulating metabolism, and this could also explain the observed phenotype. Adam's current metabolism system model does not represent kinase control mechanisms, and so Adam did not take this into account. There is also the possibility that YIL033C is both a kinase and a glutaminase, and some evidence exists to support this theory (see [31]).

See the Robot Scientist website for more details on Adam's results, its hardware and its software (see appendix note 4).

##### Formalisation

We formalised the information related to Adam's investigations. This was based on the generic ontology of scientific experiments: EXPO [32,33]. We developed a custom version of EXPO called LABORS (see appendix note 5) which was tailored to formalise Adam's experiments. We also developed an ontology to describe experimental actions (both by humans and machines) called EXACT [34].

LABORS was developed when no generic formalism for the logical description of experiments was available. The OBI project aims to provide such a formalism and the first release is due in the near future [35]. The Robot Scientist project joined the OBI consortium in October 2008, and the LABORS representations which are common to other biological domains have been aligned with the OBI representations. However, other terms specific to automated investigations still remain within LABORS. Use of LABORS resulted in the generation of a nested tree structure 10 levels deep containing over 10,000 research elements, connecting all the experimental information to the observations. These data are expressed in the logical programming language Datalog [36], and have been made publicly available (see the *'Results' *section on the Robot Scientist website - see appendix note 4). This explicit logical description makes Adam's investigations more explicit, reproducible, and reusable.

#### A Robot Scientist to study chemical genetics and drug design - 'Eve'

**Figure 4:**
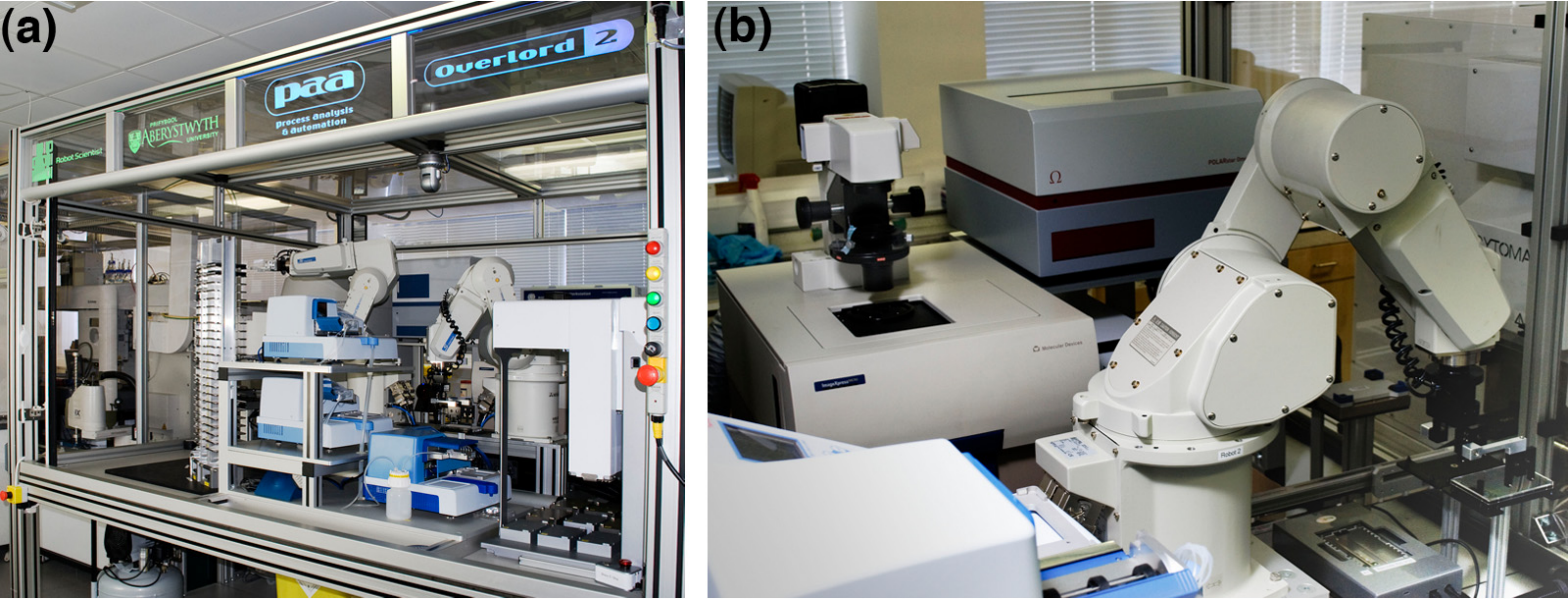
**Eve's laboratory robotic system**. (a) An external view of Eve's laboratory robotic system, also showing Adam's at the extreme left, and (b) a view looking down on some of the instruments within Eve's robotic system.

Our second Robot Scientist, Eve, was physically commissioned in the early part of 2009 (see Figure 4). Both the software and the biological assays are still under development. Eve is a prototype system to demonstrate the automation of closed-loop learning in drug-screening and design. Eve's robotic system is capable of moderately high-throughput compound screening (greater than 10,000 compounds per day) and is designed to be flexible enough such that it can be rapidly re-configured to carry out a number of different biological assays.

The main goal with Eve is to integrate machine learning and automated quantitative structure-activity relationship (QSAR [37]) into the drug-screening process, to improve both the efficiency and quality, as well as reduce the cost, of a primary drug screen. Eve will begin by performing a standard mass screen against the target assay, monitoring the results in real time, and when sufficient hits are found it will stop the mass screen. After verifying the hits Eve will then switch to a more targeted approach using machine learning and QSARs to look at the chemical structures of the hit compounds, and generate hypotheses about what it considers would be useful compounds to test next. It then plans the screening experiments to test these hypotheses, runs these experiments on the robotic system, uses machine learning to analyse these results, and then iteratively cycles around testing other compounds until it can identify the best set of lead compounds for the target. Eve will first test those compounds which are available from its own compound library, then suggest other compounds that are commercially available that should be tested. Potentially Eve could even suggest new compounds that should be synthesised for testing.

Eve will initially use an automation-accessible compound library of 14,400 chemical compounds: the Maybridge 'Hit-finder' library (see appendix note 6). This compound library is cluster-based and was developed specifically to contain a diverse range of compounds. It was selected as a subset of the full Maybridge compound library by a two-stage filtering process based first on 'Lipinski's rule of five' [38] to reduce the set to 200,000 compounds, then secondly by using a Pharmacophore Fingerprinting process [39] and cluster analysis to further reduce the set to 14,400 compounds. We realise that this is not a large compound library by industrial standards; a pharmaceutical company may have many hundreds of thousands or even millions of compounds in its primary screening library. Our aim is to demonstrate the proof-of-principle that incorporating machine learning and QSARs into the screening process can improve on the current mass screening approach.

**Figure 5:**
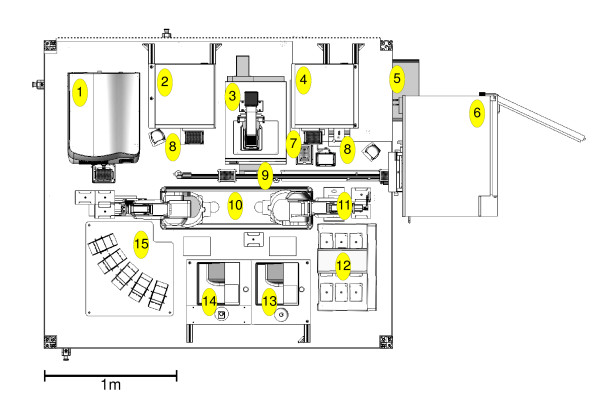
**Plan diagram of Eve's laboratory robotic system**. Layout diagram of Eve's laboratory robotic system, comprising: [1] Labcyte Echo 550 acoustic liquid handler, [2] BMG Pherastar reader, [3] MDS ImageXpress Micro cellular imager, [4] BMG Polarstar reader, [5] Cytomat 2C435 incubator, [6] Cytomat 6003 dry store, [7] FluidX DC-96pro capper/recapper, [8] two Variomag teleshake plate shakers and two Metrologic Orbit 1D barcode readers, [9] Cytomat linear actuator track, [10] robot plinth holding two Mitsubishi robot arms; models RV-3SJB and RV-3SJ, [11] FluidX Xtr-96 tube rack 2D barcode scanner, [12] Agilent (Velocity 11) Bravo liquid handler, [13] Thermo Combi-nL multidrop, [14] two Thermo Combi multidrops, and [15] consumables stacks for microplates, tube racks and tips. There are also two computers controlling the robotics, plus a networked computer server which runs all the other code vital to Eve's function: the chemistry knowledge base, QSARs and hypothesis generation, experiment planning, results relational database, data analysis etc. (not shown).

Eve's laboratory robotic system (see Figure 5) contains a carefully selected set of instruments designed to give the system the flexibility to prepare and execute a broad variety of biological assays, including: cellular growth assays, cell based chemical compound screening assays, and cellular morphology assays. There are three types of liquid handling instruments included in the system, one of which uses advanced non-contact acoustic transference (see appendix note 7), as used by many large pharmaceutical companies. For observation of assays, the system contains two multi-functional microplate readers (see appendix note 8) capable (with the appropriate filters) of recording measurements across a broad range of both excitation and emission wavelengths. There is also an automated cellular imager (see appendix note 9) capable of taking images of the well contents of microplates using both bright-field and a broad range of other wavelengths. This automated high-throughput microscope could be used to collect cell morphological information, for example to see how cells change size and shape over time after the addition of specific compounds. Also, the primary biological assays intended to be used on Eve will create one or more fluorescent protein markers that can be detected on the readers and imager, such that Eve can not only quantify the amount of marker produced using the readers, but also potentially localise it to specific cellular regions or organelles using the imager. Eve also utilises control software for the robotic system that is flexible enough to allow us to reconfigure the experimental process (see appendix note 10). In all, we believe this system is equivalent to the best systems available in the Pharmaceutical industry.

In addition, Eve is also physically connected to Adam via a linear track slide which allows the transfer of microtitre plates in either direction. This will allow development of assays using equipment from both systems and further increases the flexibility available in experimental design.

##### Eve's software

As with our other Robot Scientist Adam, Eve is also intended to be fully automated. Eve will include software components that will allow the system to perform cycles of targeted drug screening. There are three stages to this approach; mass screening, hit verification, and hypothesis-driven targeted screening. For each of these stages there will be experiment design code that generates the biological experiment plans, which combine chimeric yeast target strains and chemical compounds.

First, the mass screening experiment plans will be generated and passed to the robotic system control software (see appendix note 10) for execution. Monitoring software will automatically analyse the mass screening data in real time to identify and quantify chemical 'hits', and when there are sufficient hits, the system will be switched into the verification mode.

Experiment generation code will then create the verification microplate layouts to re-screen each hit against the target at multiple concentrations and with multiple replicates. Once these have been executed on the robotic system, the resulting dose-response curves will be analysed by curve smoothing algorithms and statistical tests to create a refined list of verified hits that includes quantitative information about how each chemical affected the target.

This list will then be passed to machine learning code that uses the quantitative information and QSARs to inspect the chemical structures of the hits and create hypotheses about other possibly active compounds. Cycles of targeted screening then commence to test these hypotheses, with the experiment planning code generating microplate layouts and executing them on the robotic system. The aim is for the machine learning code to analyse each successive cycle of this targeted screening and progressively refine an optimal list of lead compounds.

Eve will use the same relational database that was developed for Adam to store all its data and meta-data, with some modifications specific to the drug discovery research and storing additional instrument meta-data.

##### Formalisation of Eve

As with Adam, the intention is to formalise all data and meta-data relating to Eve, again by creating a derivation of the EXPO ontology. There are many similarities between Adam and Eve's data and meta-data, as well as in the types of instruments used on the systems, so we plan to identify the common elements in both the LABORS and new Eve ontologies, and update EXPO to include this common understanding.

### The Future

The immediate future for our Robot Scientist work involves the continued development of the new Eve system and the improvement and continued use of the Adam system. Additionally, the two systems will be capable of working together to address scientific questions. By combining the functionality of the instrumentation on both systems we will be able to discover more about yeast, bacteria and *C. elegans *by preparing experiments that measure a variety of phenotypes including growth, fluorescent protein expression, cellular morphology, chemical susceptibility, growth competition, multiple gene deletions, and other visible phenotypic assays. For example, Adam's robotic system could prepare plates containing multiple cellular strains that could then be passed to Eve's robotic system where multiple compounds could be added before visualisation of the morphological effects using the automated cellular imager. The longer-term aim is for Robot Scientists to become more commonplace in the laboratory: automating reasoning, decision making and information management, as well as automating the execution of experimental procedures. We believe that such automation is necessary to meet the challenges of 21st century biological research.

## Discussion

Any automated scientific discovery system or Robot Scientist clearly has both advantages and disadvantages. Some of the main points are discussed here.

It has been suggested that systems such as ours would be better described as 'Laboratory Assistants' rather than the implicitly more independent term 'Scientists'. Although in some ways the term 'Laboratory Assistant' has some merit, as they are not independent workers. In other, more important ways, the term is inappropriate as Laboratory Assistants do not generally form hypotheses, decide on the experiments to test them, automatically analyse and interpret the results etc. It should also be remembered that these systems are still just prototypes, and it is probable that future developments in hardware and software will increase the independent nature of such systems. So, on balance, we prefer the more evocative term 'Robot Scientist', and argue that the Adam system has discovered new knowledge about gene function in *S. cerevisiae *that has been independently verified [24].

Another common argument against Robot Scientists is that they remove the chance for serendipitous discovery, and that they are incapable of innovation. We would argue that more often than not a serendipitous discovery is simply the result of an experiment that has been designed without prior analysis of all the potential outcomes. Louis Pasteur phrased this sentiment as *'In the fields of observation chance favours only the prepared mind'*. While it is true that the underlying artificial intelligence components fail to meet human expectations for innovative thought, we believe that developing a richer background model and incorporating more sophisticated reasoning mechanisms will bring us closer to that goal.

It is also true that a Robot Scientist generates its hypotheses based on information obtained from publicly available databases, and as such is susceptible to any errors contained therein. However, this is no different to the situation in which human scientists find themselves, as they also have to rely on published information. Most such databases are curated by humans, and provide a service that biologists routinely use in their work. Both could choose (or in the case of the Robot Scientist be programmed) to assign weightings in their confidence of various pieces of evidence based on how they were labelled in the databases (e.g. indirect vs direct experimental evidence). When errors are present the robot is most likely to propose an incorrect hypothesis which the experimental data will then refute. For Adam we avoided problems by primarily using only one public database (KEGG) and manually updating our system model where conflicts were noticed, before allowing automated generation of hypotheses. Where a Robot Scientist may have more trouble is where it lacks the broader background knowledge base which may be available to a human scientist (e.g. the problem with Adam's system model not representing kinases mentioned earlier). A better system model and a broader knowledge base can be developed for Robot Scientists that in time would negate this difference.

Similarly, it has been pointed out that the data analysis algorithms of a Robot Scientist might be less able to deal with flaws in experimental measurements than a human, and may come to false conclusions as a consequence. We believe this to be mostly a matter of refinement of programming; for example, Adam's growth curve smoothing and de-noising routines use machine learning and statistical data analysis of multiple replicates to routinely deal with the effects of significant noise, contamination, and even gaps in the measured readings. Further refinement could be done to identify abnormal or other unexpected results, for example the shape of a bacterial contamination growth curve, or the long lag time associated with yeast cross-contamination, and then discount them automatically. The advantage of a Robot Scientist here is that it would always be consistent in its handling of the data. More physical issues with an experiment, for example flaws in plastic wares, faults in instruments, incorrect placements of plates in instruments etc. are currently easier for a human to notice and correct. Whilst there are some measures we can put in place to automatically deal with this type of issue (e.g. calibration and fault detection system checks prior to experiment runs), we believe that future refinements of the hardware and plastic wares used in laboratory automation will reduce the effects of this type of problem.

Finally, there has also been discussion about costs, comparing the cost of using a Robot Scientist against using humans to perform the same tasks. There is a substantial cost for these systems, not only in initial capital outlay and user training, but also in ongoing servicing and maintenance costs, and we would not currently consider them to be 'cost-effective' in comparison to human scientists. However, these systems are early prototypes, and we would expect such costs to reduce significantly as laboratory automation becomes more widespread, more reliable, and the software more user-friendly. The cost of hiring human scientists and technicians and buying the instruments and equipment they need to perform such high-throughput and complex experiments should not be underestimated either, and the robots have the advantages of efficiency, consistent quality, and the ability to run outside normal working hours.

## Conclusions

Robot Scientists are the next logical step in laboratory automation. They can automate all aspects of the scientific discovery process: they generate hypotheses from a computer model of the domain, design experiments to test these hypotheses, run the physical experiments using robotic systems, and then analyse and interpret the results. They also have the potential to record every detail of what they have done and why, enabling scientific investigations to be more reproducible and reuseable. We look forward to a time when Robot Scientists will commonly work with human scientists to progress the path of science.

## Appendix

1. Robot Scientist informatics, http://www.aber.ac.uk/compsci/Research/bio/robotsci/data/informatics/

2. Sciclone i1000, Caliper Life Sciences, Hopkinton, MA, USA

3. Spectramax 190 readers, MDS Analytical Technologies, Concord, Ontario, Cananda

4. Robot Scientist website, at http://www.aber.ac.uk/en/cs/research/cb/projects/robotscientist/

5. LABORS ontology: http://www.aber.ac.uk/compsci/Research/bio/robotsci/data/data/LABORS.owl

6. Maybridge Hitfinder library, Maybridge, Cornwall, UK

7. Echo 550 acoustic liquid handler, Labcyte, Sunnyvale, CA, USA

8. Pherastar and Polarstar readers, BMG labtech BmgH, Offenburg, Germany

9. ImageXpress Micro, MDS Analytical Technologies, Concord, Ontario, Cananda

10. Overlord 2 control software, PAA Ltd., Farnborough, UK

## Competing interests

The authors declare that they have no competing interests.

## Authors' contributions

Areas of work and authors listed alphabetically:

AI software (EB, KEW); automation hardware and control software(AC, AS, JR); automation requirements specification (AC, AS, KEW, JR, LNS, MY, WA); biology and manual experiments (MM, MY, WA); chemical compound definition (MNK); growth curve analysis (JR, ML, MY); hypothesis generation and bioinformatics (KEW, ML); logical formalisation of data (AC, LNS, ML); ontologies (LNS); procurement (JR); relational database (AC, AS, MNK); statistics and data analysis (AC, AS, ML); writing this manuscript (AC, AS, JR, ML); yeast metabolism model (KEW).

RDK conceived the idea, led the project and was involved in all aspects of it.

All authors read and approved the final manuscript.

## References

[B1] Langley P The computer-aided discovery of scientific knowledge Lecture Notes in Computer Science Proceedings of the First International Conference on Discovery Science 1998 1532 25 39

[B2] Džeroski S Todorovski L (Eds) Computational Discovery of Scientific Knowledge: Introduction, Techniques, and Applications in Environmental and Life Sciences 2007 Berlin, Heidelberg: Springer-Verlag

[B3] Szalay A Gray J 2020 Computing: Science in an exponential world Nature 2006 440 7083 413 414 10.1038/440413a 16554783

[B4] Lindsay R Buchanan B Feigenbaum E Lederberg J DENDRAL: A Case Study of the First Expert System for Scientific Hypothesis Formation Artificial Intelligence 1993 61 2 209 261 10.1016/0004-3702(93)90068-M

[B5] Lenat DB Brown JS Why AM and EURISKO appear to work Artificial Intelligence 1984 23 3 269 294 10.1016/0004-3702(84)90016-X

[B6] Kulkarni D Simon H The processes of scientific discovery: The strategy of experimentation Cognitive Science: A Multidisciplinary Journal 1988 12 2 139 175 10.1207/s15516709cog1202_1

[B7] Langley P Simon H Scientific discovery: Computational explorations of the creative processes 1987 The MIT press

[B8] Falkenhainer B Michalski R Integrating quantitative and qualitative discovery: the ABACUS system Machine Learning 1986 1 4 367 401

[B9] Zytkow J Automated discovery of empirical laws Fundamenta Informaticae 1996 27 2-3 299 318

[B10] Nordhausen B Langley P A robust approach to numeric discovery Proceedings of the seventh international conference (1990) on Machine learning 1990 Morgan Kaufmann Publishers Inc. San Francisco, CA, USA 411 418

[B11] Schmidt M Lipson H Distilling Free-Form Natural Laws from Experimental Data Science 2009 324 5923 81 85 10.1126/science.1165893 19342586

[B12] Muggleton S 2020 computing: exceeding human limits Nature 2006 440 7083 409 410 10.1038/440409a 16554781

[B13] Dittrich P Manz A Lab-on-a-chip: microfluidics in drug discovery Nature Reviews Drug Discovery 2006 5 3 210 218 10.1038/nrd1985 16518374

[B14] Yamashita T Nishimura I Nakamura T Fukami T A System for LogD Screening of New Drug Candidates Using a Water-Plug Injection Method and Automated Liquid Handler Journal of the Association for Laboratory Automation 2009 14 2 76 81 10.1016/j.jala.2008.10.001

[B15] Hogan C Simons S Zhang H Burdick D Living with Irresolute Cell Lines in an Automated World Journal of the Association for Laboratory Automation 2008 13 3 159 167 10.1016/j.jala.2008.01.004

[B16] Saitoh S Yoshimori T Fully Automated Laboratory Robotic System for Automating Sample Preparation and Analysis to Reduce Cost and Time in Drug Development Process Journal of the Association for Laboratory Automation 2008

[B17] Manley J Smith T Holden J Edwards R Liptrot G Modular approaches to automation system design using industrial robots Journal of the Association for Laboratory Automation 2008 13 13 23 10.1016/j.jala.2007.09.003

[B18] Maccio M Bell D Davolos D Modular Automation Platforms: A Case Study of a Flexible NMR Sample Preparation Robot Journal of the Association for Laboratory Automation 2006 11 6 387 398 10.1016/j.jala.2006.10.007

[B19] Foster I 2020 Computing: A two-way street to science's future Nature 2006 440 7083 419 10.1038/440419a 16554785

[B20] Whelan K King R Using a logical model to predict the growth of yeast BMC Bioinformatics 2008 9 97 10.1186/1471-2105-9-97 18269749 PMC2335308

[B21] Flach P Kakas A Ray O Abduction, Induction, and the Logic of Scientific Knowledge Development Workshop on Abduction and Induction in AI and Scientific Modelling 2006 Citeseer

[B22] Michalski R Watanabe L Constructive Closed-Loop Learning: Introductory Ideas and Examples Tech. rep., Tech. Rep. No. MLI-Report 88-1 1988 Fairfax, VA: George Mason University, Artificial Intelligence Center

[B23] King R Whelan K Jones F Reiser P Bryant C Muggleton S Kell D Oliver S Functional genomic hypothesis generation and experimentation by a robot scientist Nature 2004 427 247 252 10.1038/nature02236 14724639

[B24] King R Rowland J Oliver S Young M Aubrey W Byrne E Liakata M Markham M Pir P Soldatova L Sparkes A Whelan K Clare A The Automation of Science Science 2009 324 5923 85 89 10.1126/science.1165620 19342587

[B25] Pouliot Y Karp P A survey of orphan enzyme activities BMC bioinformatics 2007 8 244 10.1186/1471-2105-8-244 17623104 PMC1940265

[B26] Reiser P King R Kell D Muggleton S Bryant C Oliver S Developing a logical model of yeast metabolism Electronic Transactions in Artificial Intelligence 2001 5 223 244

[B27] Muggleton S De Raedt L Inductive logic programming: Theory and methods Journal of Logic Programming 1994 19/20 629 679 10.1016/0743-1066(94)90035-3

[B28] Muggleton S Inverse entailment and Progol New generation computing 1995 13 3 245 286 10.1007/BF03037227

[B29] Kakas A Kowalski R Toni F Abductive logic programming Journal of logic and computation 1992 2 6 719 10.1093/logcom/2.6.719

[B30] Breiman L Random forests Machine learning 2001 45 5 32 10.1023/A:1010933404324

[B31] Cannon J Gitan R Tatchell K Yeast cAMP-dependent protein kinase regulatory subunit mutations display a variety of phenotypes Journal of Biological Chemistry 1990 265 20 11897 11904 2164021

[B32] Soldatova L Clare A Sparkes A King R An ontology for a Robot Scientist Bioinformatics 2006 22 14 10.1093/bioinformatics/btl207 16873508

[B33] Soldatova L King R An ontology of scientific experiments Journal of the Royal Society Interface 2006 3 11 795 803 10.1098/rsif.2006.0134 PMC1885356 17015305

[B34] Soldatova L Aubrey W King R Clare A The EXACT description of biomedical protocols Bioinformatics 2008 24 13 i295 10.1093/bioinformatics/btn156 18586727 PMC2718634

[B35] The OBI Consortium SIG: Bio-Ontologies: Modeling biomedical experimental processes with OBI Proceedings ISMB/ECCB 2009 2009 Oxford University Press

[B36] Ullman J Principles of database and knowledge-base systems, Classical Database Systems 1988 I Computer Science Press, Inc. New York, NY, USA

[B37] Dudek A Arodz T Gálvez J Computational methods in developing quantitative structure-activity relationships (QSAR): a review Combinatorial Chemistry and High Throughput Screening 2006 9 3 213 10.2174/138620706776055539 16533155

[B38] Lipinski C Lombardo F Dominy B Feeney P Experimental and computational approaches to estimate solubility and permeability in drug discovery and development settings Advanced Drug Delivery Reviews 1997 23 1-3 3 25 10.1016/S0169-409X(96)00423-1 11259830

[B39] Butina D Unsupervised data base clustering based on daylight's fingerprint and Tanimoto similarity: A fast and automated way to cluster small and large data sets Journal of Chemical Information and Computer Sciences 1999 39 4 747 750

